# Patient Risk Factor Profiles Associated With Timing of Goals-of-Care Consultation Before Death

**DOI:** 10.1093/geroni/igaa057.3043

**Published:** 2020-12-16

**Authors:** Lauren Starr, Connie Ulrich, Paul Junker, Liming Huang, Nina O’Connor, Salimah Meghani

**Affiliations:** 1 University of Pennsylvania School of Nursing, Philadelphia, Pennsylvania, United States; 2 University of Pennsylvania Health System, Philadelphia, Pennsylvania, United States; 3 University of Pennsylvania, Philadelphia, Pennsylvania, United States

## Abstract

Early palliative care consultation to discuss goals-of-care (“PCC”) benefits seriously ill patients. To identify risk factor profiles associated with inpatient PCC timing before death, we conducted a secondary analysis of seriously ill adults who had PCC at a high-acuity hospital and died 2014-2016. Of 1,141 patients, 54% had PCC “close to death” (0-14 days before death); 26% had PCC 15-60 days before death; 21% had PCC >60 days before death (median 13 days). Classification and Regression Tree modeling showed Hispanic or “Other” race/ethnicity intensive care patients with extreme illness severity (85%) were most likely to have PCC close to death, with age <46 or >75 increasing probability (98%). Among age groups, the highest proportion of patients with PCC close to death was >75 years. Complex variable interactions associated with PCC timing suggests we need a systematic process for initiating PCC earlier and effective primary palliative training for providers across settings.

